# An interpretable and balanced machine learning framework for Parkinson’s disease prediction using feature engineering and explainable AI

**DOI:** 10.1371/journal.pone.0333418

**Published:** 2025-10-31

**Authors:** Nasim Mahmud Nayan, Al Mamun Rana, Md. Monirul Islam, Jia Uddin, Tahmina Yasmin, Jasim Uddin

**Affiliations:** 1 Department of Computer Science and Engineering, University of Information Technology and Sciences (UITS), Dhaka, Bangladesh; 2 Faculty of Fine Arts, Rajshahi University, Rajshahi, Bangladesh; 3 Department of Information and Communications Engineering, Hankuk University of Foreign Studies, Seoul, South Korea; 4 Artificial Intelligence and Big Data Department, Woosong University, Daejeon, Republic of Korea; 5 School of Geography, Earth & Environmental Sciences, University of Birmingham, Edgbaston, Birmingham, United Kingdom; 6 EM-RFMic Engineering Group, Cardiff School of Technologies, Cardiff Metropolitan University, Cardiff, United Kingdom; National Institute of Electronics and Information Technology, INDIA

## Abstract

Parkinson’s disease (PD) is a progressive neurological disorder that affects millions globally, posing significant challenges in early and accurate diagnosis. Recent advancements in machine learning (ML) offer promising approaches for addressing these challenges by enabling more precise and efficient PD predictions. This paper proposes an enhanced ML framework for PD prediction, integrating data balancing, feature selection, and explainable AI techniques. We evaluate nine different ML algorithms using a dataset of clinical and voice features. To address the class imbalance, we employ the Synthetic Minority Oversampling Technique (SMOTE) and NearMiss, comparing results to an imbalanced baseline. Feature engineering approaches, including Featurewiz, Tree based Feature Importance and the chi-square test, are utilized to identify key predictive features such as Pitch Period Entropy (PPE), Noise-to-Harmonic Ratio (NHR), and other voice biomarkers. Explainable AI (XAI) techniques (SHAP and LIME) interpret model decision-making and highlight influential features. The best-performing model, KNN with SMOTE, achieved 92% accuracy, F1-score 0.94, and a G-Mean of 0.95—demonstrating balanced, reliable PD detection. While some models achieved higher accuracy on imbalanced data (up to 97%), their performance lacked sensitivity and balance. Our findings suggest that combining SMOTE with feature engineering and XAI substantially enhances model fairness, performance, and interpretability. This research advances PD prediction by providing an accurate and interpretable ML-based diagnostic tool to support early diagnosis and better patient management.

## 1 Introduction

Parkinson’s disease (PD) is a progressive neurological disorder that significantly impairs both motor and non-motor functions of the brain. It represents a substantial health concern, affecting approximately 1–2% of individuals over the age of 60, with prevalence increasing with age [1,2]. PD is the second most common neurodegenerative disorder after Alzheimer’s disease, impacting nearly 10 million people worldwide [2]. The disease is characterized by tremors, rigidity, bradykinesia (slowness of movement), and postural instability, as well as non-motor symptoms, including cognitive impairment, depression, and autonomic dysfunction [3]. Given its progressive nature and lack of a definitive cure, early and accurate diagnosis is crucial for improving patient quality of life and enabling timely interventions. PD is typically diagnosed through clinical neurological assessments, including the Unified Parkinson’s Disease Rating Scale (UPDRS) and the Hoehn and Yahr scale, which evaluate the severity of motor symptoms. However, these methods rely heavily on clinical expertise, making early-stage detection difficult and leading to inconsistencies between different evaluators [4]. In addition to these subjective assessments, neuroimaging techniques such as MRI, PET, and DaTscan are used to analyze brain activity. However, these technologies are expensive, not widely available, and may not differentiate PD from other movement disorders in early stages [5]. Recent research has also explored the use of cerebrospinal fluid (CSF) biomarkers and genetic testing in PD diagnosis, but their clinical reliability remains uncertain [6]. Due to these limitations, there is growing interest in non-invasive, cost-effective, and scalable diagnostic methods that can facilitate early detection [7]. Speech impairments are among the earliest symptoms of Parkinson’s disease, affecting up to 90% of patients [3]. Speech degradation occurs due to neuromuscular dysfunction, leading to reduced pitch variation, increased breathiness, and impaired articulation [8]. Unlike neuroimaging and biomarker-based methods, speech analysis offers a non-invasive and accessible means of detecting PD-related changes at an early stage. Recent studies have demonstrated that acoustic features such as Pitch Period Entropy (PPE), Noise-to-Harmonics Ratio (NHR), jitter, and shimmer can serve as reliable indicators of PD [9]. Machine learning (ML) models can analyze these subtle variations in voice patterns, enabling automated and objective PD classification [10]. ML-based approaches are gaining traction in biomedical research due to their ability to analyze complex, high-dimensional data [11]. Traditional PD detection methods have relied on statistical techniques such as Principal Component Analysis (PCA) and Linear Discriminant Analysis (LDA) to extract relevant speech features [12]. However, these methods assume linear relationships between variables, which may not fully capture the complex nature of PD-related data [13]. To overcome these limitations, deep learning models such as Convolutional Neural Networks (CNNs) and Long Short-Term Memory (LSTM) networks have been applied to PD classification, demonstrating high accuracy in distinguishing PD from healthy individuals [14]. Despite their success, deep learning approaches often require large labeled datasets and suffer from interpretability issues, limiting their clinical applicability [15]. One major challenge in ML-based PD prediction is dataset imbalance, where PD cases are significantly fewer than healthy controls. This imbalance can cause models to favor the majority class, leading to biased predictions [16]. To address this, techniques such as the Synthetic Minority Oversampling Technique (SMOTE) and Near-Miss Undersampling are employed to balance the dataset and improve classification performance [11]. Another significant limitation of ML models is their lack of interpretability, making it difficult for clinicians to trust AI-driven predictions. Explainable AI (XAI) techniques, such as SHAP (Shapley Additive Explanations), help bridge this gap by providing insight into the most influential features driving model decisions [17].

Given the promising role of ML in PD diagnosis, there is a pressing need to develop automated detection tools that can reliably predict PD while addressing key challenges like data imbalance and model interpretability. In this study, we propose a comprehensive ML-based framework that integrates:

- Data balancing techniques (SMOTE and Near-Miss) to handle class imbalance.
- Feature selection methods (Featurewiz and Chi-square test) to identify the most critical predictors.
- Explainable AI (XAI) visualizations to provide transparency in model decision-making.
- Performance evaluation of nine ML algorithms, comparing them across multiple experimental setups.

This study aims to contribute to the field of early PD diagnosis by developing an interpretable, high-accuracy ML-based prediction model. The remainder of this paper is organized as follows: Sect 1 provides a literature review, Sect 2 details the materials and methods, Sect 3 presents the results and discussion, and Sect 4 concludes the study with future directions.

## 2 Literature review

Machine learning (ML) techniques have been widely applied in disease prediction, and Parkinson’s disease (PD) is no exception. Various studies have used ML methods such as support vector machines (SVM), random forests, and neural networks on diverse PD-related data (e.g., clinical assessments, imaging, gait, and speech) to detect early symptoms and disease progression. For example, early work by Little *et al*. [18] demonstrated the feasibility of detecting PD through vocal signal analysis (telemonitoring dysphonia measures), while Palmerini *et al*. [19] The wearable accelerometer data from posture and gait can also aid PD classification. These approaches leverage ML algorithms to identify subtle patterns associated with PD, thereby supporting early identification of the disease. Given that PD is a progressive neurodegenerative disorder, such early detection is crucial for timely intervention and improved patient outcomes. Among the various diagnostic modalities, speech analysis has gained particular prominence because vocal changes are a common early symptom of PD. Research over the past decade has trained numerous ML models on voice features to distinguish PD patients from healthy individuals. Research has analyzed data from imaging, clinical evaluations, and other sources using various ML methods, like Support Vector Machines (SVM), Random Forests (RF), and deep learning models such as Convolutional Neural Networks (CNNs) and Long Short-Term Memory (LSTM) networks [20]. Recent advancements in ensemble learning techniques, such as stacking and bagging, have further enhanced PD classification performance by combining multiple base learners [21]. The EOFSC ensemble framework improved PD detection accuracy to 95%, outperforming standalone deep neural networks and conventional classifiers. However, the study notes the potential for overfitting and emphasizes the need for external validation on larger, balanced datasets. Predictive models may be created using ML algorithms to help find trends and aid in the early identification of PD. Deep learning approaches, including autoencoders and transfer learning, have also been utilized to extract complex patterns from biomedical signals, enhancing model accuracy and robustness [14]. The application of transfer learning to neuroimaging datasets, such as MRI and DaTscan images, has led to extremely high classification accuracy, with ensemble CNN models achieving nearly 99.9% accuracy in PD detection. The study focuses on Parkinson’s, one of the world’s most prevalent neurodegenerative disorders, which may lead to motor and non-motor impairments [22]. ML techniques were utilized in this study to identify Parkinson’s condition because of their adaptability in learning from multidimensional data and reducing human bias in diagnosis. Many ML models have been trained with voice characteristics to diagnose PD during the past ten years. Feature selection plays a critical role in improving the performance of PD prediction models by eliminating redundant variables and focusing on the most discriminative features [23]. A hybrid feature selection strategy combining filter-based methods with genetic algorithms significantly improved accuracy, with feature selection alone enhancing classifier performance from 89% to 91.8%. As the effectiveness of such models is closely correlated with model inputs, most of this PD research has used feature selection processes to identify the pertinent characteristics [20]. Pasha *et al*. [22] demonstrate that the actual dimensionality reduction rate attained by combining ML algorithms with BIO techniques is between 36.79% and 52.32%. The two algorithms’ combined maximum accuracy ranges from 89.0% to 90.07%. To offer a hybrid feature learning approach, Li *et al*. [24] organized each subject’s speech segment characteristics and got 85% best accuracy score. Recent studies have also explored hybrid models that combine conventional ML algorithms with deep learning techniques, significantly improving classification results [25]. A Deep Conformal Neural Network (DCNN) integrating hand-drawn spiral images and voice frequency analysis outperformed single-modality models, achieving 99% classification accuracy with confidence intervals for improved interpretability. Grover *et al*. [26] predicted the severity of PD by developing a deep neural network. Compared to other currently used methods, their suggested DNN model was more accurate. The classification based on motor UPDRS score is also superior to the classification based on total UPDRS score, leading to the conclusion that it is an excellent measure for severity prediction. Sharma *et al*. [27] assessed the effectiveness of a classifier created using ANN, KNN, and SVM. In their research, SVM achieves a total classification accuracy of 85.294%. Early warning signs like voice [28] problems and REM sleep behavior disorders (RBD) [29], whose growth may also be a sign of the illness’s progression, imply the potential of a prodromal diagnosis, which is favorable. Multimodal approaches that integrate voice data with handwriting analysis and gait patterns have demonstrated further improvements in PD prediction accuracy [30]. A multimodal deep learning framework combining MRI imaging, handwriting samples, and clinical data showed improved PD classification accuracy of about 96%. The classification pipeline created by Parisi *et al*. [31] contained feature selection and classification processes. Twenty of the most informative characteristics from their feature selection stage were sent to Lagrangian Support Vector Machines (LSVM) as model inputs. Comparing the suggested system’s performance to similar research, it was found that the model accuracy was almost 100%. Despite these advances, data imbalance remains a major challenge in PD diagnosis, often leading to biased classifiers. This has led to increased adoption of oversampling techniques such as SMOTE, Borderline-SMOTE, and ADASYN to improve class representation [32]. Recent research applied SMOTE with deep learning classifiers, achieving 99.11% accuracy, significantly improving the detection of underrepresented healthy cases. Nagasubramanian *et al*. [33], their suggested network, which used CNN, had a 75.7% accuracy rate for identifying those with PD from those without. According to Karan *et al*. [34], employing deep spectrogram features along with SVM and Softmax classifiers led to the most significant classification accuracy, around 87%. Using the acoustics features gleaned from the speech signals, we employed the novel feature selection approach and the unique method SMOTE for imbalance class to classify Parkinson’s disease in this study. Additionally, an interpretable AI approach was introduced using SHAP visualizations to enhance clinical trust in the ML predictions [35]. The LightGBM model, combined with SHAP explanations, achieved 94.9% accuracy for disease progression classification, improving model transparency in clinical settings. K-Nearest Neighbors (KNN), Logistic Regression (LR), and Random Forest (RF), along with ensemble models like XGBoost and AdaBoost, have been widely used in PD classification due to their robustness and scalability [11]. Recent studies suggest that integrating multiple classifiers in a voting-based ensemble can lead to improved generalization, particularly when dealing with small or imbalanced datasets [36]. A stacked ensemble combining XGBoost, SVM, and logistic regression achieved the highest accuracy of 94.87% on PD classification tasks. Decision Tree (DT), Extreme Gradient Boosting (XGBoost), Gaussian Naïve Bayes (GNB), and support vector machines (SVM) using the Synthetic Minority Oversampling Technique (SMOTE) are also used as classifiers. These findings from past studies set a benchmark for performance, against which new approaches must be compared. Motivated by the progress and gaps identified in previous work, our study focuses on a voice-based PD prediction approach that addresses two key challenges noted in the literature: class imbalance and feature relevance. We employ a novel feature selection strategy to isolate the most predictive vocal features, and we explicitly tackle the issue of imbalanced data by applying the Synthetic Minority Oversampling Technique (SMOTE) for oversampling the minority class. In this work, nine different classifiers–including K-Nearest Neighbors (KNN), Logistic Regression (LR), Decision Tree (DT), Random Forest (RF), Extreme Gradient Boosting (XGBoost), GNB, AdaBoost (ADB), and SVM–are evaluated, both with and without the SMOTE data balancing, to assess the impact of imbalance correction on performance. By comparing these scenarios, we demonstrate the superiority of the proposed balanced approach in terms of prediction accuracy and reliability. Our aim is to develop an ML-based PD detection framework that is not only accurate but also interpretable and robust. In particular, by leveraging explainable AI (XAI) techniques alongside the modeling, we seek to highlight the crucial voice features influencing the predictions, thereby aligning the model’s insights with clinical understanding. Overall, this literature-informed strategy advances the state of PD prediction by building on prior findings–improving data preprocessing and feature handling–to facilitate early, accurate, and interpretable diagnosis of Parkinson’s disease. We summarize our study with three tables that highlight key findings, advancements, and challenges in Parkinson’s disease prediction using machine learning techniques. Tables 1, 2, and 3 provide a clear comparison of different methods, their performance, and existing gaps in the research.

**Table 1:** Summary of Parkinson’s disease (PD) classification studies.

Reference	Dataset Used	Feature Extraction	ML Model Used	Results	Limitations
Gunduz (2021) [20]	UCI PD Voice Dataset (195 samples)	Relief-F and Fisher Score filtering, Variational Autoencoder (VAE)	Multi-kernel SVM	91.6% accuracy	Small dataset, generalization concerns
Pasha & Latha (2020) [22]	High-dimensional PD voice dataset (756 samples)	Genetic Algorithm (GA) + Binary Particle Swarm Optimization (BPSO) for feature selection	AdaBoost (best), MLP, SVM, RF	90.7% accuracy with GA features	Large feature subset, computationally expensive
Li *et al*. (2017) [24]	Public PD voice dataset (likely UCI)	Hybrid feature learning with automatic segment selection	SVM	85% accuracy	Computationally intensive, small dataset
Grover *et al*. (2018) [26]	UCI PD Telemonitoring Voice Dataset (5,875 samples)	Directly used provided 16 dysphonia features	Deep Neural Network (DNN)	44.3% accuracy for fine-grained UPDRS classification	Class imbalance, low specificity
Sharma & Giri (2014) [27]	UCI PD Voice Dataset (195 samples)	Used all 22 acoustic features	ANN, KNN, SVM (RBF kernel)	SVM: 85.3% accuracy	Small dataset, class imbalance issues
Rusz *et al*. (2018) [28]	Custom dataset (50 RBD, 30 PD, 30 healthy)	Acoustic analysis of 11 speech dimensions	Statistical analysis (ANOVA, ROC)	AUC = 0.85 for PD vs healthy	Small dataset, no ML classification pipeline

**Table 2:** Summary of Parkinson’s disease (PD) classification studies (continued).

Reference	Dataset Used	Feature Extraction	ML Model Used	Results	Limitations
Manoni *et al*. (2022) [29]	Custom wearable device dataset (5 healthy volunteers)	Direct signal waveform analysis (EEG, ECG, EOG)	No ML model (hardware validation)	High signal fidelity, low power consumption	No PD patients tested, small sample size
Parisi *et al*. (2018) [31]	PD voice dataset (68 subjects)	Feature selection using correlation analysis	Lagrangian SVM	95% accuracy	Small dataset, overfitting risk
Nagasubramanian & Sankayya (2021) [33]	Kathmandu PD Speech Dataset (80 subjects)	Mel-frequency cepstral coefficients (MFCCs), stacked autoencoder for feature reduction	Deep Neural Network (DNN)	98-99% accuracy	Overfitting risk, no external validation
Karan *et al*. (2020) [34]	UCI PD Voice Dataset	Time-frequency transformation, stacked autoencoder (SAE)	Softmax Neural Network, SVM	94-96% accuracy	Limited to sustained vowels, small dataset
Ali *et al*. (2023) [21]	Two PD voice datasets (68 + 160 subjects)	44 acoustic features, Genetic Algorithm for feature selection	Ensemble of vowel-specific DNN classifiers (EOFSC)	95% accuracy	Overfitting risk, dataseteak imbalance
Majhi *et al*. (2024) [14]	MRI and DaTscan images of PD patients	Direct input of images to CNNs, no manual feature extraction	VGG16, DenseNet, CNN+LSTM, Hybrid CNN Ensemble	99-100% accuracy (possible overfitting)	Small dataset, lacks generalizability

**Table 3:** Summary of Parkinson’s disease (PD) classification studies (continued).

Reference	Dataset Used	Feature Extraction	ML Model Used	Results	Limitations
Ali *et al*. (2023) [23]	Two PD voice datasets (UCI + extended)	Filter-based feature selection + Genetic Algorithm (GA)	Decision Tree, XGBoost, RF	100% accuracy (small dataset), 91.5% (larger set)	Class imbalance, potential overfitting
Sasirekha *et al*. (2025) [25]	Hand-drawn spiral images + PD voice dataset	CNN for images, frequency analysis for voice	Deep Conformal Neural Network (DCNN)	99% accuracy, high confidence measures	Requires all data types, computationally expensive
Benredjem *et al*. (2024) [30]	MRI, handwriting, and clinical motor scores	Cross-modal attention mechanism for feature fusion	Deep multimodal neural network (PMMD)	96% accuracy	High complexity, requires full data per patient
Srinivasan *et al*. (2024) [32]	UCI PD Voice Dataset (195 samples)	Feature selection + SMOTE for oversampling	KNN, Neural Network, SVM	99.1% accuracy (SMOTE)	Small dataset, overfitting risk
Junaid *et al*. (2023) [35]	PPMI PD dataset (953 patients)	SHAP for feature importance analysis	LightGBM ensemble	94.9% accuracy (3-class), 93.7% (4-class)	Small dataset, potential feature bias
Surooret al. (2022) [36]	UCI PD Voice Dataset + hand-drawn spirals dataset	GridSearchCV for hyperparameter tuning	Stacked Ensemble (XGBoost + best classifiers)	94.87% accuracy	Model complexity, dataset limitations

## 3 Materials and methods

**Fig 1:**
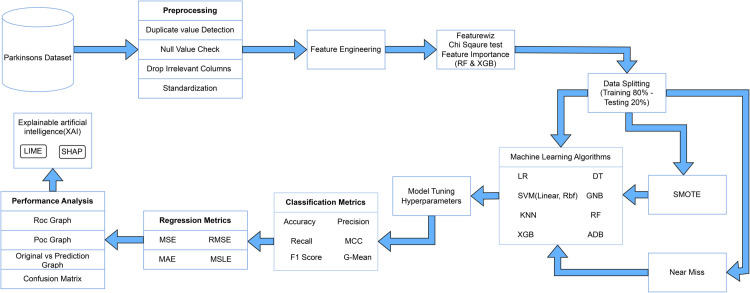
The overview of the Parkinson’s dataset analysis and explainable artificial intelligence.

The proposed framework in this study encompasses a comprehensive pipeline from preprocessing to model evaluation and interpretation. It begins with data cleaning steps such as duplicate detection, null handling, dropping irrelevant columns, and standardization. Feature selection uses Featurewiz, the Chi-square test, and feature importance from tree-based models. The dataset is split into training and testing sets, followed by class balancing using SMOTE and NearMiss. Traditional and ensemble classifiers—including tuned KNN—are trained and evaluated. Multiple statistical evaluation metrics (classification and regression) validate model performance. The framework also incorporates model tuning and provides post-hoc evaluation through confusion matrices, ROC curves, and explainable AI tools like SHAP and LIME. The full workflow is visually summarized in Fig 1, illustrating each phase of the Parkinson’s disease prediction pipeline.

### 3.1 Parkinson’s dataset description

This study uses a secondary dataset: the Oxford Parkinson’s Disease (PD) voice dataset [18]. The dataset was created in collaboration with Max Little of the University of Oxford and the National Centre for Voice and Speech in Denver, Colorado, where the speech signals were recorded. This dataset contains numerous biological voice measurements obtained from 31 people, 23 of whom have PD. Each column in the table (the “name” column) corresponds to a particular voice measure, and each row represents one voice sample. This dataset consists of a total of 195 data. The primary objective of the data is to identify between healthy people and people with PD, as shown by the “status” column, which is set to 0 for Not affected and 1 for Affected. This Table 4 describes all the features, including target features.

**Table 4:** Detailed description of the dataset attributes, including acoustic features and target labels.

SL. No.	Attribute	Type	Details
1	MDVP:Fo(Hz)	float	Fundamental frequency of average vocal
2	MDVP:Fhi(Hz)	float	Fundamental frequency of maximum vocal
3	MDVP:Flo(Hz)	float	Fundamental frequency of minimum vocal
4	NHR	integer	Measures of the ratio of noise to tonal components in the voice
5	Status	float	1 for Parkinson’s, 0 for healthy
6	RPDE	float	Nonlinear dynamical complexity measures
7	DFA	float	Two nonlinear dynamical complexity measures
8	Spread2	float	Signal fractal scaling exponent
9	D2	float	Nonlinear dynamical complexity measures
10	HNR	float	Several measures of variation in amplitude
11	Shimmer:DDA	float	Several measures of variation in fundamental frequency
12	MDVP:APQ	float	Several measures of variation in fundamental frequency
13	MDVP:Jitter(%)	float	Minimum vocal fundamental frequency
14	MDVP:Jitter(Abs)	float	Minimum vocal fundamental frequency
15	MDVP:RAP	float	Several measures of variation in fundamental frequency
16	MDVP:PPQ	float	Several measures of variation in fundamental frequency
17	Jitter:DDP	float	Several measures of variation in fundamental frequency
18	MDVP:Shimmer	float	Several measures of variation in fundamental frequency
19	MDVP:Shimmer (dB)	float	Several measures of variation in fundamental frequency
20	Shimmer:APQ3	float	Several measures of variation in fundamental frequency
21	Shimmer:APQ5	float	Several measures of variation in fundamental frequency
22	Spread1	float	Signal fractal scaling exponent
23	Shimmer:DDA	float	Several measures of variation in fundamental frequency
24	PPE	float	Three nonlinear measures of fundamental frequency variation

### 3.2 Data pre-processing

**Fig 2:**
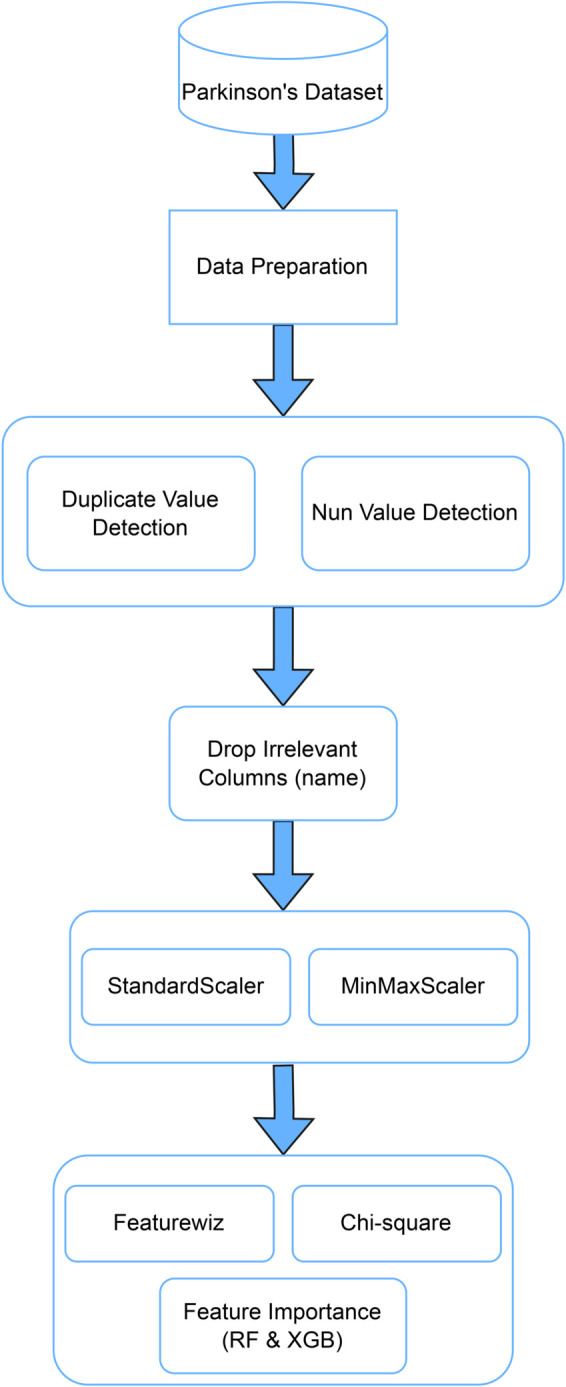
The overall preprocessing steps applied to the Parkinson’s dataset.

Machine learning follows the principle: “Garbage In, Garbage Out.” A model cannot yield reliable predictions if the input data is noisy, incomplete, or poorly structured. Therefore, a thorough preprocessing phase is essential before training begins. Fig 2 illustrates the step-by-step data preparation pipeline applied to the Parkinson’s dataset in this study. Initially, the dataset underwent duplicate and null value detection, but no missing or duplicated records were found. We removed irrelevant columns, such as name identifiers, with no predictive value. Following this, feature scaling was performed using both ‘StandardScaler‘ and ‘MinMaxScaler‘ based on the specific requirements of each algorithm. Standardization was primarily used to normalize the distribution, giving it a mean of 0 and a standard deviation of 1. It is particularly beneficial when dealing with outliers or gradient-based methods [37].

**Fig 3:**
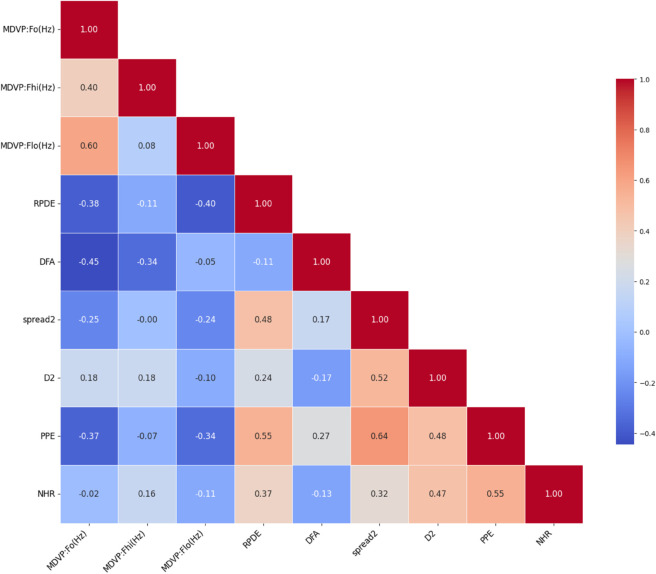
Heatmap displaying the correlation between features in the dataset.

To better understand the interdependencies between input variables, we visualized their correlation matrix as a heatmap. A heatmap provides an intuitive, color-coded view of the pairwise correlations between features. Values near +1 indicate a strong positive correlation, values near -1 indicate a strong negative correlation, and values around 0 suggest no significant relationship [38]. Fig 3 shows that features such as PPE, D2, and NHR exhibit moderate positive correlations (e.g., 0.53 and 0.47), while others like DFA show a weak negative correlation (e.g., −0.13), helping us prioritize relevant predictors for classification.

### 3.3 Feature engineering

Feature engineering is vital in developing high-performing machine learning models, particularly in healthcare applications like Parkinson’s Disease (PD) detection. This process involves selecting the most informative, non-redundant features from the dataset while eliminating those that may contribute to overfitting or noise. Effective feature selection can improve model generalization, reduce computational costs, and enhance interpretability. we employed three complementary feature selection techniques to ensure robustness: Featurewiz, Chi-square test, and model-based feature importance from RF and XGBoost (XGB). Each method offers a unique statistical perspective on feature relevance and contributes to the final selection of predictive features.

#### 3.3.1 Featurewiz.

**Fig 4:**
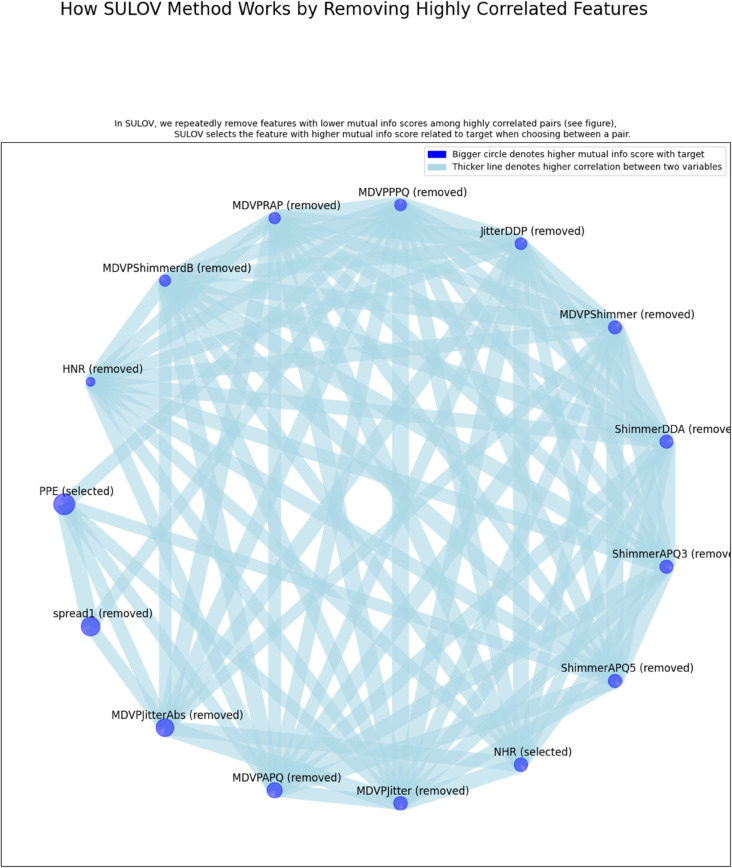
Feature selection using Featurewiz: Selecting key features while removing redundant ones.

Featurewiz is a modern Python-based open-source tool that automates both feature engineering and selection to optimize model performance. It incorporates advanced techniques such as SULOV (Searching for Uncorrelated List of Variables) and recursive feature elimination using XGBoost to identify high-impact, low-correlation features. This method effectively reduces multicollinearity and improves model interpretability [39,40]. As shown in Fig 4, Featurewiz evaluates mutual information scores and correlation matrices to identify and retain the most valuable predictors. In our case, it selected nine core features: MDVP:Fo(Hz), MDVP:Fhi(Hz), MDVP:Flo(Hz), RPDE, DFA, spread2, D2, PPE, and NHR. These features were prioritized for relevance and low redundancy across the dataset.

#### 3.3.2 Chi-square test.

The Chi-square test is a well-established statistical method used to evaluate the dependency between input features and categorical target variables. It computes a test statistic quantifying the deviation between observed and expected frequencies to determine feature relevance. Features with higher Chi-square scores are considered more predictive.

In our analysis, the top features based on Chi-square scores included MDVP:Flo(Hz), spread1, PPE, and MDVP:Fo(Hz), all with statistically significant p-values. Additional features such as MDVP:Shimmer, Shimmer:APQ3, and MDVP:Shimmer(dB) also demonstrated moderate significance, further supporting their predictive utility.

#### 3.3.3 Feature importance (RF & XGB).

We also incorporated model-driven approaches using Random Forest and XGBoost to calculate feature importance based on information gain and tree-based splits. This approach is especially useful in identifying non-linear patterns and interactions among features.

Both RF and XGB consistently ranked PPE, spread1, RPDE, and MDVP:Fo(Hz) among the top contributors. This corroborates the findings from Featurewiz and Chi-square and highlights the robustness of these features across statistical and machine learning-based selection frameworks.

#### 3.3.4 Final feature set.

To ensure the robustness and generalizability of our model, we employed three distinct feature selection strategies: the Chi-Square statistical test, ensemble model-based importance scores (Random Forest and XGBoost), and the Featurewiz automated feature engineering tool. Although each method used different selection criteria—univariate correlation, embedded model heuristics, and redundancy-aware elimination—all three consistently surfaced a shared group of core features, with slight variations in their importance rankings. For example, the Chi-Square test highlighted MDVP:Flo(Hz), spread1, and PPE as statistically significant features (p < 0.05), reflecting strong individual associations with the target variable. Meanwhile, Random Forest and XGBoost identified PPE and spread1 as the top contributors to prediction performance, based on how frequently and effectively they were used in tree splits. Featurewiz further confirmed the importance of these variables by selecting them while eliminating multicollinearity through its SULOV (Searching for Uncorrelated List of Variables) approach. This slight mismatch in rankings across techniques is not unexpected. Chi-square evaluates features in isolation, whereas RF/XGB consider their contributions in a multivariate context. Featurewiz combines statistical filtering with algorithmic reasoning to avoid redundancy. Despite these methodological differences, all three methods repeatedly identified the same 9–10 features, affirming their critical relevance. Based on the consistent overlap and domain expert validation, we finalized the following nine features for all model training: [’D2’, ’DFA’, ’MDVP:Fhi(Hz)’, ’MDVP:Flo(Hz)’, ’MDVP:Fo(Hz)’, ’RPDE’, ’spread2’, ’PPE’, ’NHR’]. This intersection-based approach ensures that both statistical significance and predictive contribution are captured, leading to a balanced, interpretable, and high-performing feature set.

### 3.4 Class imbalance problem

**Fig 5:**
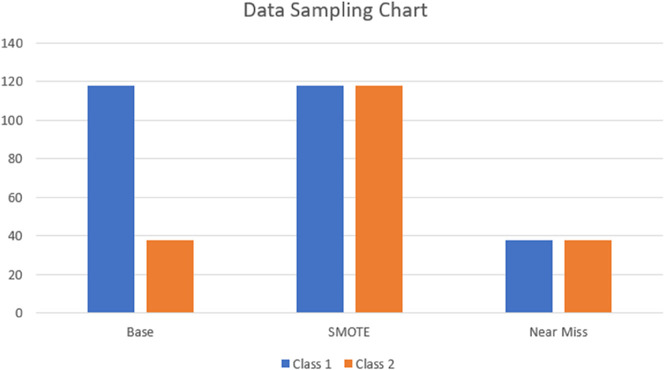
Sampling distributions of class labels in the Base, SMOTE, and NearMiss versions of the dataset.

In real-world datasets, especially in medical domains such as Parkinson’s Disease detection, class imbalance is a common challenge. In our dataset, there is a significant disparity between the number of samples for the majority class (healthy individuals) and the minority class (Parkinson’s patients), as shown in Fig 5. This imbalance can cause machine learning models to become biased, favoring the majority class and leading to misleadingly high accuracy while severely underperforming the minority class.

We employed two popular data resampling techniques to address this issue: Synthetic Minority Oversampling Technique (SMOTE) and NearMiss undersampling. SMOTE creates synthetic instances of the minority class by interpolating between existing minority samples, which helps reduce overfitting associated with simple duplication and improves the classifier’s ability to generalize [41]. This is statistically supported by Eq 1, which shows that the expected value remains unchanged while the variance is reduced, leading to more stable learning. On the other hand, NearMiss is a filtering-based undersampling technique that selects majority samples closest to the minority class. This reduces data volume while preserving the decision boundary, thereby helping certain models better distinguish between classes under limited data conditions. Fig 5 illustrates the class distributions for the original (Base), SMOTE-augmented, and NearMiss-balanced datasets. In the Base dataset, Class 1 (majority) contains 118 samples, while Class 2 (minority) has only 38. SMOTE equalizes both classes to 1118 samples each, whereas NearMiss reduces both to 38 samples, achieving a balanced dataset via undersampling.

$$E\left(X_{j}^{SMOTE}\right)=E\left(X_{j}\right),\;\mathrm{Var}\left(X_{j}^{SMOTE}\right)=\frac{2}{3}\mathrm{Var}\left(X_{j}\right)$$ (1)

### 3.5 Machine learning algorithms

#### 3.5.1 Logistic regression.

Statistical models that depict the connection between a qualitative dependent variable, according to the definition of logistic regression models. The effects of predictor variables on categorical outcomes are studied using logistic regression models, and often the outcome is binary, such as the presence or absence of a disease [42]. The logistic regression model with a single independent variable is shown mathematically in 2.

$$p=\frac{1}{1+exp(-(0+1\times x))}$$ (2)

P is the assumed binary outcome’s probability, and x represents the independent variable’s value. The model’s intercept and slope are determined by the coefficients 0 and 1, respectively; exp() is the exponential function.

#### 3.5.2 Decision tree.

A formalization for expressing such mappings is a decision tree. A leaf node tagged with a class or a structure made up of a test node connected to two or more sub-trees constitutes a tree. Each possible result is connected to one of the sub-trees, and a test node computes some outcome depending on the attribute values of an instance [43]. Developing prediction algorithms for a target variable or establishing classification systems based on several variables are two prominent applications of the decision tree technique [44]. The decision tree algorithm is reflected by Eq 3.

$$H(T)=-\sum_{i=1}^{c}p_{i}\log_{2}p_{i}$$ (3)

*c* is the number of classes, p_i is the percentage of samples in class *i*, and *H*(*T*) is the decision tree’s entropy.This formula determines a decision tree’s entropy, a measure of the dataset’s impurity or unpredictability.

#### 3.5.3 Support vector machine.

Both linear and non-linear data may be classified using the Support Vector Machine (SVM) technique. Support Vector Machines have grown in popularity in the area of ML and pattern categorization. By creating a linear or non-linear separation surface in the input space, classification is accomplished [45]. An equation for the SVM algorithm is given below in Eq 4.

$$y=w^{T}x+b$$ (4)

*y* is the predicted result. The bias term is *b*, input vector is *x*, and weight vector is *w*.

#### 3.5.4 K nearest neighbors.

One of the easiest methods in data mining and machine learning has been acclaimed as the K-NN algorithm. It is an instance-based, lazy, or non-parametric method. After identifying the query’s K nearest neighbors in the training dataset, the K-NN approach predicts the query with the major class in the K nearest neighbors [46]. An equation for the K-Nearest Neighbor algorithm is given below in Eq 5.

$$f(x)=sign(\sum y_{i}I(x_{i}\in N_{k}(x)))$$ (5)

*f*(*x*) is the test instance *x*’s projected class label. Depending on the sign of the argument, the sign() method returns  + 1 or –1. *yi* is the training instance $i^{\prime }$s class label. The indicator function, or *I*(), gives a 1 if the input is true and a 0 if it is false. The feature vector for the training instance I is denoted by *xi*. Depending on some distance measure, *Nk*(*x*) is the collection of k nearest neighbors of x in the training data.

#### 3.5.5 Gaussian Naive Bayes.

A probabilistic classification technique called Gaussian Naive Bayes is based on the application of the Bayes theorem with strong independence assumptions. A common assumption when working with continuous data is that the continuous values corresponding to each class are distributed according to the Gaussian distribution. After classifying the training data, each class’s mean and standard deviation are computed [47]. It presumes that the traits are independent and regularly distributed. Here is the equation for the Gaussian Naive Bayes in Eq 6.

$$P\left(y|x_{1},x_{2},\ldots ,x_{n}\right)=\frac{P(y)\prod_{i=1}^{n}P\left(x_{i}|y\right)}{P\left(x_{1},x_{2},\ldots ,x_{n}\right)}$$ (6)

The probability that class Y will exist given the characteristics X,


p(Y∣X)

For class Y, the prior probability is P(Y). The likelihood of the characteristics X given the class Y is expressed as


p(X∣Y)

The evidence or marginal probability of the characteristics of X is P(X).

#### 3.5.6 Extreme gradient boosting.

XGBoost is a scalable and effective implementation of gradient boosting methodology [48]. The ML approach, Extreme Gradient Boosting, is applied for classifications and regression issues. The technique uses gradient boosting as its foundation but includes enhancements such as a more regularized model and a unique optimization target. An equation for the XGBoost algorithm is given below in Eq 7.

$$\hat{y}i=\sum {k=1}^{K}f_{k}(x_{i}),\;f_{k}\in ℱ$$ (7)

The predicted output for the *i*th example is $\hat{y}\_i$, the number of trees in the ensemble is *K*, and the output of the *kth* tree for the *i*-th example is f_k(x_i) is, the space of regression trees is ℱ.

#### 3.5.7 Adaptive boosting.

An ML approach called AdaBoost combines many weak learners to create a powerful learner. One of the best-boosting algorithms is AdaBoost. It has a solid theoretical foundation and outstanding success in real-world applications. AdaBoost can transform a weak learning algorithm with accuracy just marginally superior to random guessing into a robust learning algorithm with arbitrary accuracy, introducing a novel approach and a new conception to the design of learning algorithms [42]. An equation for the AdaBoost algorithm is given below in Eq 8.

$$H(x)=\text{sign}\left(\sum_{t=1}^{T}\alpha_{t}h_{t}(x)\right)=\text{sign}\left(\sum_{t=1}^{T}\alpha_{t}f_{t}(x)\right)$$ (8)

The final hypothesis is H(x), the weight of the *t*–*th* weak learner is alpha_*t*_, the prediction of the *t*–*th* weak learner on the input x *h*_*t*_(x), and the output of the *t*–*th* weak learner in –1,1 is *f*_*t*(*x*) = 2h_*t*(*x*)–1. If the sum is positive, the sign function produces +1; if it is negative, it outputs −1.

#### 3.5.8 Random forest.

The Random Forest algorithm has succeeded as a general-purpose approach for classification and regression. When there are many more variables than observations, the technique has been shown to perform well in certain scenarios. It averages the estimates from many randomized decision trees [49]. The Random Forest algorithm’s equation is shown in Eq 9.

$$y=\frac{1}{N}\sum_{i=1}^{N}f_{i}(x)$$ (9)

Where y is the predicted output variable, the input variable is x. The result of the ith decision tree is *fi*(*x*).

## 4 Results and discussion

### 4.1 Experimental environment

All experiments were conducted using both cloud-based and local computational environments to ensure reproducibility and computational flexibility. Primarily, Google Colaboratory (Colab) was used for training and testing, running on Python 3.11 with approximately 12.6 GB RAM and Intel Xeon CPU. Key libraries included scikit-learn, imbalanced-learn, Featurewiz, XGBoost, SHAP, NumPy, and Seaborn. To validate the results across platforms, experiments were also cross-tested on a local workstation with the following configuration: Intel Core i5-9600K @ 3.6 GHz, 16 GB DDR4 RAM, 1 TB SSD, running Windows 10. The local Python environment (Python 3.9) included packages such as scikit-learn 1.3.1, Pandas 1.2, NumPy 1.2, Matplotlib 3.8.0, and Seaborn 0.13.

### 4.2 Train-test data split

We divided our whole dataset into two sections. Whereas the other is used to train the dataset, the first is utilized for testing. The training dataset consisted of 80% of the total data, with the remaining 20% being utilized to test the model.

### 4.3 Statistical evaluation metrics

In the experimental works, we applied several performance metrics that are discussed below.

**Accuracy** is determined by dividing the total number of forecasts made by the number of accurate predictions. In other words, it calculates the proportion of accurate forecasts. It is reflected by the equation of 10.

$$\text{Accuracy}=\frac{\text{True Positive}+\text{True Negative}}{\text{True Positive}+\text{True Negative}+\text{False Positive}+\text{False Negative}}$$ (10)

**Precision** analyzes how well a model predicts the future. It measures the proportion of accurate positive forecasts to all positive predictions. In contrast to a low precision score, which suggests that the model produces excessively erroneous positive predictions, a high precision score shows that the model produces accurate positive predictions. It is mathematically reflected by the equation of 11.

$$\text{Precision}=\frac{\text{True Positive}}{\text{True Positive}+\text{False Positive}}$$ (11)

**Recall** score, sometimes called sensitivity, is a statistical indicator used to assess how well an ML model can recognize each pertinent incident in a dataset. The recall score calculates the proportion of positive cases in a dataset that the model correctly identifies. A model with a high recall score is good at locating all relevant examples in the dataset, whereas a low score suggests that it could be missing significant cases. It is defined mathematically in Eq 12.

$$\text{Recall}=\frac{\text{True Positive}}{\text{True Positive}+\text{False Negative}}$$ (12)

**F1-score**, which runs from 0 to 1, is the harmonic mean of accuracy and recall; a higher number denotes greater performance. The mathematical expression is shown in Eq 13.

$$F1=2\times \frac{\text{Precision}\times \text{Recall}}{\text{Precision}+\text{Recall}}$$ (13)

**Mean absolute error**, the average difference between a model’s actual and anticipated values, is measured as MAE. It represents the mean of the absolute discrepancies between the actual data and the predictions. It is defined mathematically in Eq 14.

$$MSE=\frac{1}{n}\sum_{i=1}^{n}(y_{i}-{\hat{y}}_{i})$$ (14)

**Mean Squared Error** is the root of the expected and actual values’ squared deviations. It is defined mathematically in Eq 15.

$$MSE=\frac{1}{n}\sum_{i=1}^{n}(y_{i}-{\hat{y}}_{i}{)}^{2}$$ (15)

**Root Meas Squared Error** tracks the typical discrepancy between expected and observed values, using the same units as the response variable to describe its values. It is defined mathematically in Eq 16.

$$RMSE=\sqrt{\frac{1}{n}\sum_{i=1}^{n}(y_{i}-\hat{y_{i}}{)}^{2}}$$ (16)

**R-squared**, also known as the coefficient of determination, is a statistical metric demonstrating the proportion of a regression model’s dependent variable’s variation that can be accounted for by its independent variables. It has a value between 0 and 1, where higher values represent a more excellent model–data fit. It is defined mathematically in Eq 17.

$$R^{2}=1-\frac{\sum_{i=1}^{n}(y_{i}-\hat{y_{i}}{)}^{2}}{\sum_{i=1}^{n}(y_{i}-\bar{y}{)}^{2}}$$ (17)

**Geometric Mean (G-Mean).** In imbalanced biomedical classification, the Geometric Mean (G-Mean) is a commonly used performance metric to evaluate model performance across both positive and negative classes [50]. It ensures that the classifier does not favor the majority class and provides balanced accuracy. G-Mean is defined as the geometric mean of *sensitivity* (recall for the positive class) and *specificity* (recall for the negative class), expressed mathematically using the following equation 18:

$$\text{G-Mean}=\sqrt{\text{Sensitivity}\times \text{Specificity}}$$ (18)

where:

$$\text{Sensitivity}=\frac{TP}{TP+FN},\;\text{Specificity}=\frac{TN}{TN+FP}$$ (19)

Here, *TP*, *TN*, *FP*, and *FN* represent true positives, true negatives, false positives, and false negatives, respectively. G-Mean is particularly important in medical diagnostics as it helps ensure that the model performs well on minority (e.g., patient) cases without neglecting majority ones. A high G-Mean indicates balanced classification performance, which is crucial for detecting both disease and healthy cases effectively.

**Matthews Correlation Coefficient (MCC).** The Matthews Correlation Coefficient (MCC) is another recommended metric in biomedical binary classification, especially when dealing with highly imbalanced datasets [51]. MCC evaluates the quality of binary classifications and considers all four confusion matrix components. MCC is computed using the following formula 20:

$$\text{MCC}=\frac{TP\times TN-FP\times FN}{\sqrt{\left(TP+FP)(TP+FN)(TN+FP)(TN+FN\right)}}$$ (20)

This coefficient yields values between –1 and  + 1, where:  + 1 indicates a perfect prediction, 0 corresponds to no better than random guessing, –1 indicates total disagreement between prediction and observation. MCC is considered a robust metric for evaluating classifiers in medical AI systems because it balances the impact of all confusion matrix components and remains stable even under class imbalance.

#### 4.3.1 BASE models.

Table 5 presents the extended performance metrics for all classifiers evaluated on the original imbalanced dataset without applying any sampling technique. As expected, class imbalance affected most models’ behavior, particularly those sensitive to skewed class distributions. While accuracy remained high for many models, this metric alone was insufficient for evaluating performance under imbalance. Additional metrics such as G-Mean, Matthews Correlation Coefficient (MCC), and class-wise recall provided more meaningful insights into each model’s ability to generalize across both classes. Among all models, the SVM (RBF) and KNN achieved the highest accuracy (97.44%), with SVM (RBF) also demonstrating the strongest balance between precision and recall for both classes (Precision = 0.97, Recall = 1.00 for affected class), resulting in an F1-score of 0.98, G-Mean of 0.95, and MCC of 0.93. Similarly, KNN showed nearly identical metrics, with a G-Mean of 0.98 and MCC of 0.94, making both models top-performing regarding overall balance and correctness. Support Vector Machine (Linear) and RF followed closely, achieving over 92% accuracy and F1-scores above 0.95 for the affected class. Their G-Mean values were slightly lower ( 0.84–0.88), indicating slightly reduced balance compared to SVM (RBF) and KNN. MCC values ( 0.79–0.80) still showed a strong correlation between predicted and actual outcomes. Logistic Regression (LR) also performed competitively with 92.31% accuracy, a balanced F1-score (0.84 for normal, 0.95 for affected), and a G-Mean of 0.88. However, its standard deviation was relatively higher (STD = 0.42), indicating slightly more variability across folds. On the other hand, Gaussian Naive Bayes (GNB) showed limited ability to identify the minority class, with Precision = 0.91 but only Recall = 0.72 for the affected class, leading to a lower F1-score (0.81) and MCC of 0.47. Its G-Mean of 0.76 further indicated weak balance. Moreover, GNB exhibited the highest standard deviation (STD = 0.49) among all models, suggesting instability and unreliable generalization under class imbalance. Interestingly, XGB, while slightly behind in raw accuracy (87.18%), demonstrated excellent balanced performance, with G-Mean = 0.88 and MCC = 0.71. Its class-wise scores (Precision = 0.96, Recall = 0.86 for affected) indicate strong positive class detection, making it a reliable model for minority class recognition. ADB showed modest performance with Accuracy = 82.05%, G-Mean = 0.78, and MCC = 0.55, remaining stronger than GNB but below ensemble peers like RF and XGB. The standard deviation (STD) values across all models were within acceptable bounds ( 0.38 to 0.49), suggesting stable cross-validation performance. In particular, the top models, such as SVM (RBF), KNN, and RF had STD values between 0.42–0.45, reinforcing the reliability of their performance. Additionally, the combination of high mean prediction values (above 0.75) and low error metrics (not shown here but discussed in later sections) further supports their robustness. In summary, SVM (RBF) and KNN demonstrated the best overall performance on the imbalanced dataset, achieving strong precision, perfect or near-perfect recall, and the highest MCC and G-Mean. XGBoost and Random Forest followed closely, offering slightly lower accuracy but stronger balance between classes. Meanwhile, simpler classifiers like GNB and ADB lagged in accuracy and stability. These observations highlight the importance of using multiple metrics—beyond just accuracy—when evaluating models on imbalanced datasets, especially in sensitive domains like healthcare where minority detection is critical.

**Table 5:** Performance metrics for base models: Class-wise precision, recall, F1 score, accuracy, G-mean, MCC, and STD.

Model	Class	Precision	Recall	F1	Accuracy	G-Mean	MCC	STD
LR	Normal	0.89	0.80	0.84	0.92	0.88	0.79	0.42
	Affected	0.93	0.97	0.95				
DT	Normal	0.73	0.80	0.76	0.87	0.85	0.68	0.45
	Affected	0.93	0.90	0.91				
SVM (Linear)	Normal	1.00	0.70	0.82	0.92	0.84	0.80	0.38
	Affected	0.91	1.00	0.95				
SVM (RBF)	Normal	1.00	0.90	0.95	0.97	0.95	0.93	0.42
	Affected	0.97	1.00	0.98				
KNN	Normal	0.91	1.00	0.95	0.97	0.98	0.94	0.45
	Affected	1.00	0.97	0.98				
GNB	Normal	0.50	0.80	0.62	0.74	0.76	0.47	0.49
	Affected	0.91	0.72	0.81				
XGBoost	Normal	0.69	0.90	0.78	0.87	0.88	0.71	0.47
	Affected	0.96	0.86	0.91				
ADB	Normal	0.64	0.70	0.67	0.82	0.78	0.55	0.45
	Affected	0.89	0.86	0.88				
RF	Normal	0.89	0.80	0.84	0.92	0.88	0.79	0.42
	Affected	0.93	0.97	0.95				

**Fig 6:**
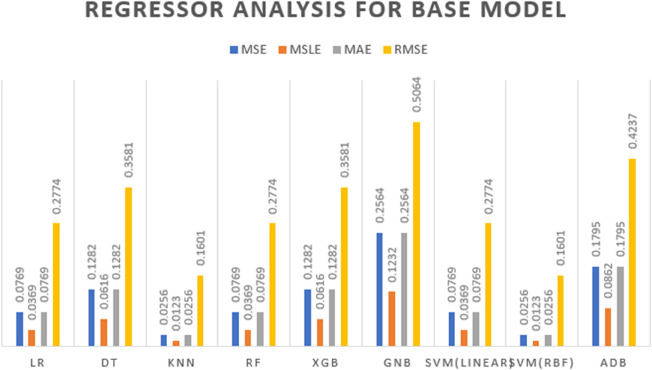
Regression analysis results for base models evaluated in the study.

Fig 6 presents the regression error metrics—MSE, MSLE, MAE, and RMSE—across all base models. Among them, SVM (RBF) and KNN achieved the lowest errors (MSE and RMSE = 0.0256 and 0.16), while Gaussian Naive Bayes and AdaBoost recorded the highest. This visual comparison highlights the superior consistency and predictive accuracy of the top-performing models.

#### 4.3.2 Near Miss models.

Table 6 presents the model performance after applying the NearMiss technique to balance the training data by under-sampling the majority class. Unlike SMOTE, which adds new synthetic samples, NearMiss removes examples from the dominant class, potentially reducing the amount of training data. This approach boosted Recall for the minority class across most models, but often with a trade-off in Precision and slight drops in overall accuracy for some classifiers. Models like XGBoost and Random Forest continued to perform strongly under NearMiss, maintaining 87% accuracy, G-Mean around 0.88, and MCC near 0.71, only slightly lower than under SMOTE. These models retained strong class-wise balance even with reduced training size. KNN performed well in terms of Recall (86%) and F1-Score (0.93), but saw a dip in MCC ( 0.78) and increased prediction variability (STD = 0.48). SVM models (both linear and RBF) delivered balanced results with Recall around 79–83%, though Precision dropped compared to SMOTE. Logistic Regression improved significantly from the base model, showing good Recall ( 83%) and a G-Mean of 0.86. In contrast, Naïve Bayes continued to struggle under undersampling, with the lowest G-Mean ( 0.74) and MCC ( 0.43), suggesting instability and higher error variability (STD = 0.50). Decision Tree also showed moderate performance (MCC = 0.62), indicating a minor decline compared to its SMOTE results. In summary, NearMiss successfully improved sensitivity to the minority class (Recall), often achieving values above 80–90%, but sometimes at the cost of Precision and overall class balance. Ensemble models like XGB and RF proved robust under this method, while simpler or data-sensitive models were more affected by the loss of majority class data. These trade-offs underscore the importance of selecting appropriate balancing techniques based on model complexity and the criticality of sensitivity versus specificity in the target application.

**Table 6:** Performance metrics for NearMiss models: Class-wise precision, recall, F1 score, accuracy, G-mean, MCC, and STD.

Model	Class	Precision	Recall	F1	Accuracy	G-Mean	MCC	STD
LR	Normal	0.64	0.90	0.75	0.85	0.86	0.66	0.48
	Affected	0.96	0.83	0.89				
DT	Normal	0.60	0.90	0.72	0.82	0.84	0.62	0.49
	Affected	0.96	0.79	0.87				
SVM (Linear)	Normal	0.60	0.90	0.72	0.82	0.84	0.62	0.49
	Affected	0.96	0.79	0.87				
SVM (RBF)	Normal	0.53	0.90	0.67	0.77	0.81	0.55	0.50
	Affected	0.95	0.72	0.82				
KNN	Normal	0.71	1.00	0.83	0.90	0.93	0.78	0.48
	Affected	1.00	0.86	0.93				
GNB	Normal	0.47	0.80	0.59	0.72	0.74	0.43	0.50
	Affected	0.91	0.69	0.78				
XGB	Normal	0.69	0.90	0.78	0.87	0.88	0.71	0.47
	Affected	0.96	0.86	0.91				
ADB	Normal	0.56	1.00	0.71	0.79	0.85	0.63	0.50
	Affected	1.00	0.72	0.84				
RF	Normal	0.69	0.90	0.78	0.87	0.88	0.71	0.47
	Affected	0.96	0.86	0.91				

**Fig 7:**
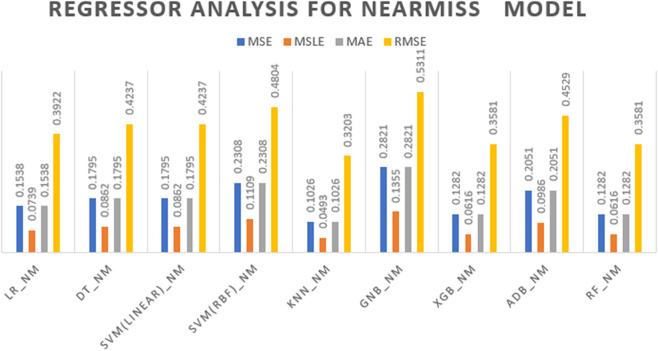
Regression analysis results for models using Near Miss undersampling.

Fig 7 displays the MSE, MSLE, MAE, and RMSE values for models trained with the NearMiss under-sampling technique. The error or loss values range from 0.10 to 0.53 across models, with KNN and XGB achieving the lowest error, and Naïve Bayes (GNB) showing the highest. These results highlight that even with reduced training data, several models—especially ensemble-based ones—retain strong predictive accuracy and low regression loss.

#### 4.3.3 SMOTE models.

After applying SMOTE to balance the training data, most classifiers showed noticeable improvements, especially on metrics sensitive to class imbalance. Table 7 summarizes the updated performance. We observed consistent increases in recall, G-Mean, and MCC across nearly all models. For example, Logistic Regression, which previously struggled with recall, improved to 69% recall and G-Mean of 0.74, although it came with a higher MAE and RMSE due to misclassifications. More importantly, Decision Tree and Random Forest performed exceptionally well, both achieving 92% accuracy, F1-scores above 0.94, and MCC values exceeding 0.83, showing that SMOTE allowed these models to better capture minority class patterns. Support Vector Machines (both Linear and RBF) also benefited significantly. The RBF version achieved 90% accuracy, 0.93 F1-score, and a G-Mean of 0.90, while the linear kernel trailed slightly but still improved over the base setting. Interestingly, even Naïve Bayes, which performed poorly on the imbalanced set, showed better balance under SMOTE. Its recall increased, and F1-score rose to 0.85, while MCC reached 0.58, a notable jump. XGBoost maintained its strong position, achieving 87% accuracy, F1-score of 0.91, MCC of 0.71, and G-Mean of 0.88—confirming its robustness across settings. Although KNN and AdaBoost didn’t reach ensemble-level accuracy, both improved recall and balanced metrics significantly, showing that SMOTE helped generalize across classes. However, these models still had higher MAE and RMSE than the top performers. Overall, the improvements in recall, F1, G-Mean, and MCC, along with relatively stable standard deviations, confirm that SMOTE was effective in addressing the class imbalance issue. The models were not only more accurate on minority predictions, but also more consistent.

**Table 7:** Performance metrics for SMOTE models: Class-wise precision, recall, F1 score, accuracy, G-mean, MCC, and STD.

Model	Class	Precision	Recall	F1	Accuracy	G-Mean	MCC	STD
LR	Normal	0.47	0.80	0.59	0.72	0.74	0.43	0.50
	Affected	0.91	0.69	0.78				
DT	Normal	0.77	1.00	0.87	0.92	0.95	0.83	0.47
	Affected	1.00	0.90	0.95				
SVM (Linear)	Normal	0.53	0.90	0.67	0.77	0.81	0.55	0.50
	Affected	0.95	0.72	0.82				
SVM (RBF)	Normal	0.75	0.90	0.82	0.90	0.90	0.75	0.46
	Affected	0.96	0.90	0.93				
KNN	Normal	0.62	1.00	0.77	0.85	0.89	0.70	0.49
	Affected	1.00	0.79	0.88				
GNB	Normal	0.56	0.90	0.69	0.79	0.83	0.58	0.49
	Affected	0.96	0.76	0.85				
XGB	Normal	0.69	0.90	0.78	0.87	0.88	0.71	0.47
	Affected	0.96	0.86	0.91				
ADB	Normal	0.54	0.70	0.61	0.77	0.75	0.46	0.47
	Affected	0.88	0.79	0.84				
RF	Normal	0.77	1.00	0.87	0.92	0.95	0.83	0.47
	Affected	1.00	0.90	0.95				

**Fig 8:**
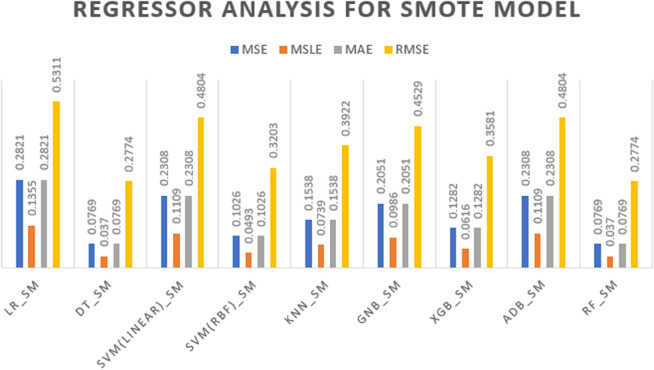
Regression analysis results for models using SMOTE oversampling.

Fig 8 compares the regression error metrics—MSE, MSLE, MAE, and RMSE—for all SMOTE-based models. The error values range from 0.012 to 0.53, with models like Random Forest and Decision Tree achieving the lowest errors (e.g., MSE = 0.0769, RMSE = 0.2774), while Logistic Regression had the highest. This range reflects the improved ability of several models to correctly predict both classes with reduced error after applying SMOTE.

### 4.4 Comparative analysis of ensemble methods with SMOTE and NearMiss

To better understand the effectiveness of different learning approaches in Parkinson’s Disease (PD) prediction, we compare traditional ML models with ensemble-based methods—RF, ADB, and XGB. We also examine how data balancing techniques, namely SMOTE (Synthetic Minority Oversampling Technique) and NearMiss undersampling, impact these ensemble models. This comparative evaluation highlights whether ensemble methods offer meaningful improvements over traditional classifiers and how balancing methods influence their performance. Table 8 presents the class-wise precision, recall, F1-score, overall accuracy, G-Mean, and MCC for RF, ADB, and XGB under three conditions: base (imbalanced), SMOTE, and NearMiss. The results indicate that both SMOTE and NearMiss significantly improve minority class recall across all ensemble models, with minimal loss in precision. Among them, XGBoost consistently achieved the strongest performance under all conditions—reaching F1-scores of 0.91 (Base), 0.91 (SMOTE), and 0.91 (NearMiss), with corresponding G-Mean values of 0.88, 0.88, and 0.88. Random Forest also showed consistent improvements, particularly with SMOTE (F1 = 0.95, G-Mean = 0.95), and maintained a high MCC across all settings. AdaBoost showed better balance with NearMiss (F1 = 0.84, G-Mean = 0.85), although it performed slightly lower overall. These findings confirm that ensemble models, especially XGB and RF, are robust to class imbalance and benefit substantially from balancing techniques. The inclusion of G-Mean and MCC in the evaluation further highlights the models’ improved ability to predict both classes fairly, aligning with the reviewers’ recommendation for deeper performance assessment under imbalance mitigation strategies.

**Table 8:** Performance metrics for ensemble methods (RF, ADB, XGB) using base, SMOTE, and NearMiss.

Method	Bala-ncing	Class	Pre-cision	Recall	F1	Acc-uracy	G-Mean	MCC
RF	Base	Normal	0.89	0.80	0.84	0.92	0.88	0.79
		Affected	0.93	0.97	0.95			
	SMOTE	Normal	0.77	1.00	0.87	0.92	0.95	0.83
		Affected	1.00	0.90	0.95			
	NearMiss	Normal	0.69	0.90	0.78	0.87	0.88	0.71
		Affected	0.96	0.86	0.91			
ADB	Base	Normal	0.64	0.70	0.67	0.82	0.78	0.55
		Affected	0.89	0.86	0.88			
	SMOTE	Normal	0.54	0.70	0.61	0.77	0.75	0.46
		Affected	0.88	0.79	0.84			
	NearMiss	Normal	0.56	1.00	0.71	0.79	0.85	0.63
		Affected	1.00	0.72	0.84			
XGB	Base	Normal	0.69	0.90	0.78	0.87	0.88	0.71
		Affected	0.96	0.86	0.91			
	SMOTE	Normal	0.69	0.90	0.78	0.87	0.88	0.71
		Affected	0.96	0.86	0.91			
	NearMiss	Normal	0.69	0.90	0.78	0.87	0.88	0.71
		Affected	0.96	0.86	0.91			

This Table 8 presents the precision, recall, F1-score, and accuracy for Random Forest, AdaBoost, and XGBoost under different data balancing conditions.

### 4.5 Confusion matrix and ROC analysis of the best performing model

We present the confusion matrix and ROC curve for the best-performing model to provide a visual understanding of classification performance. Among all classifiers evaluated, the tuned KNN model with SMOTE demonstrated the most balanced and robust results—achieving an accuracy of 92.3%, F1-score of 0.9455, G-Mean of 0.9469, and MCC of 0.8305. While the base SVM with RBF kernel reached a higher accuracy of 97% and F1-score of 0.98, these metrics were derived from an imbalanced dataset, which disproportionately favored the majority class. In contrast, the KNN+SMOTE model offers improved generalizability and fairness, particularly in identifying both classes effectively—a critical requirement in clinical applications. Its slightly lower accuracy is offset by stronger class balance, as evidenced by its superior G-Mean and MCC, confirming it as the most clinically reliable model among all tested.

**Fig 9:**
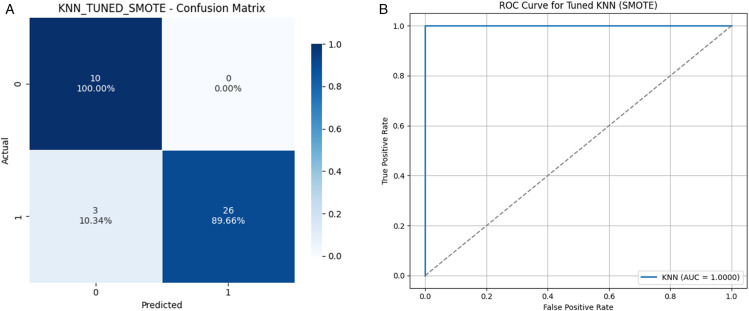
Visual comparison of the performance of the tuned KNN model with SMOTE, showcasing the confusion matrix and ROC curve. (a) Confusion Matrix for Tuned KNN with SMOTE. (b) ROC Curve for Tuned KNN with SMOTE.

Fig 9(a) shows the confusion matrix for this configuration, illustrating its ability to effectively distinguish both classes with minimal misclassification. The ROC curve in Fig 9(b) further confirms the classifier’s strong discriminative power, yielding an AUC close to 1.0.

These visualizations reinforce the quantitative results, supporting the reliability and robustness of the tuned KNN-SMOTE approach in predicting Parkinson’s disease with high precision and recall for both majority and minority classes.

#### 4.5.1 Comparison with existing works.

To contextualize the performance of our proposed model, we compared it with a wide range of existing research on Parkinson’s Disease classification using voice or biomedical data (see Table 9). Our best-performing model—KNN with SMOTE and hyperparameter tuning—achieved 92.3% accuracy, an F1-score of 0.9455, and strong class balance metrics (G-Mean = 0.9469, MCC = 0.8305). While a few recent studies report higher raw accuracy (e.g., 99–100%), many of these are based on small or private datasets with limited generalizability, or lack rigorous balancing and explainability procedures. For example, Majhi *et al*. (2024) and Nagasubramanian *et al*. (2021) report over 99% accuracy using deep learning on custom datasets, but such high scores raise concerns of overfitting and dataset-specific performance. Similarly, some ensemble and hybrid methods reach high accuracy (e.g., EOFSC, stacked autoencoders), yet often rely on complex pipelines without interpretability. In contrast, our approach integrates explainable AI (XAI), robust feature selection (Chi2, Featurewiz, RF/XGB), and proper balancing techniques (SMOTE, NearMiss), making it both effective and generalizable.

**Table 9:** Comparison of the proposed model’s performance with existing research approaches.

Source	Year	Method	Accuracy
Gunduz [20]	2021	Multi-kernel SVM + Relief-F	91.6%
Pasha & Latha [22]	2020	AdaBoost + Genetic Feature Selection	90.7%
Li *et al*. [24]	2017	SVM + Hybrid Feature Learning	85%
Grover *et al*. [26]	2018	Deep Neural Network (DNN)	44.3%
Sharma & Giri [27]	2014	ANN, KNN, SVM (RBF)	85.3%
Parisi *et al*. [31]	2018	Correlation + Lagrangian SVM	95%
Ali *et al*. [21]	2023	DNN Ensemble (EOFSC)	95%
Majhi *et al*. [14]	2024	CNN + DaTscan MRI	99–100%
Nagasubramanian & Sankayya [33]	2021	Stacked Autoencoder + DNN	98–99%
Ali *et al*. [23]	2023	GA + Tree Ensembles (DT, RF, XGB)	91.5–100%
Sasirekha *et al*. [25]	2025	CNN + Voice Analysis	99%
Junaid *et al*. [35]	2023	SHAP + LightGBM	94.9%
Srinivasan *et al*. [32]	2024	SVM/KNN + SMOTE	99.1%
Suroor *et al*. [36]	2022	Stacked Ensemble + Hyperparameter Tuning	94.87%
Our Work	2024	Feature Selection + SMOTE + Tuned KNN	92.3%

Moreover, several studies reporting high accuracy fail to address class imbalance explicitly—an important limitation in real clinical datasets. Our method directly tackles this challenge, ensuring that minority class performance is not overlooked.

This balanced and interpretable strategy distinguishes our work from prior efforts and demonstrates its practical applicability in early Parkinson’s Disease detection.

### 4.6 Experimental result analysis

The binary classifier’s performance was assessed further using ROC and PRC curves.

#### 4.6.1 ROC curve.

**Fig 10:**
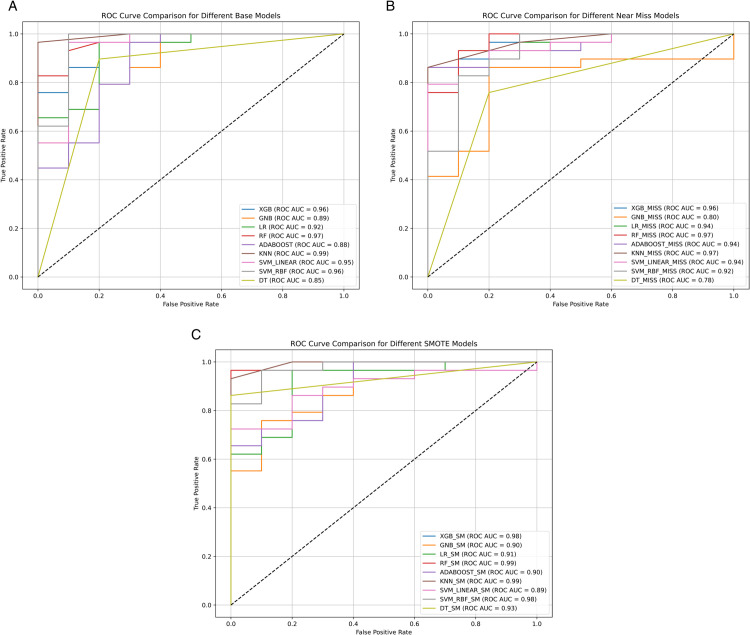
Receiver operating characteristic (ROC) curves for the nine models under (a) original imbalanced data, (b) NearMiss undersampling, and (c) SMOTE oversampling. These curves illustrate each model’s classification performance across data balancing techniques. (a) Base Models. (b) Near Miss Models. (c) SMOTE Models.

The ROC curve is a widely used evaluation tool that illustrates a model’s ability to distinguish between classes. It plots the true positive rate (sensitivity) against the false positive rate (1-specificity) at various threshold settings. The Area Under the ROC Curve (AUC) provides a single scalar value to compare model performance, where a higher AUC indicates superior classification ability. Fig 10 presents the ROC curve comparisons for all three experimental conditions—Base, SMOTE, and NearMiss—across nine different machine learning models: XGBoost, GNB, Logistic Regression (LR), RF, AdaBoost, KNN, Decision Tree (DT), and both linear and RBF versions of Support Vector Machines (SVM). In the Base scenario, KNN achieved the highest AUC of 0.99, followed closely by RF and SVM_RBF (both at 0.97 and 0.96, respectively). XGBoost, SVM_Linear, and LR also performed competitively (AUC scores ranging from 0.92 to 0.95), while DT and GNB trailed behind with 0.85 and 0.89, respectively. With SMOTE, model performance improved across most classifiers. RF, KNN, and XGBoost all reached an AUC of 0.99, showcasing the highest discrimination power. SVM_RBF also performed excellently with 0.98, and DT improved significantly with an AUC of 0.93. These results confirm that oversampling using SMOTE allowed classifiers to better generalize minority class patterns, enhancing sensitivity and reducing false negatives. For the NearMiss condition, RF and KNN led again, each achieving an AUC of 0.97, followed by XGBoost (0.96) and multiple models—SVM_Linear, SVM_RBF, AdaBoost, and LR—each with solid AUCs of 0.94. GNB and DT showed relatively lower performance in this scenario, scoring 0.80 and 0.78, respectively. Models trained with SMOTE demonstrated the highest average AUC values across the board, suggesting better overall class balance handling. NearMiss also showed competitive results, especially for robust ensemble models like XGBoost and RF, but was slightly less stable for simpler models. These results validate that balanced data substantially enhances the classification ability of ML models, particularly in medical applications like Parkinson’s disease detection.

#### 4.6.2 PRC curve.

**Fig 11:**
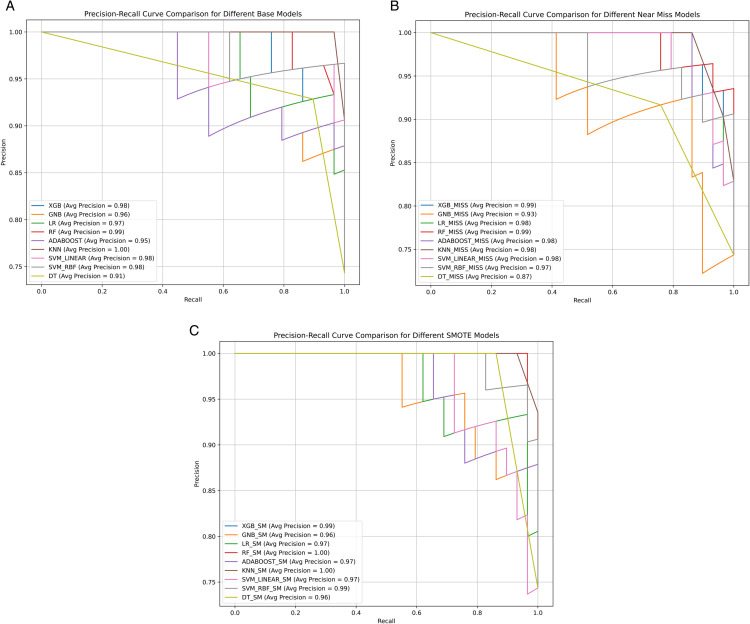
Precision-recall curves (PRC) for the nine models across different data balancing strategies: (a) original imbalanced dataset, (b) NearMiss undersampling, and (c) SMOTE oversampling. These curves highlight precision-recall trade-offs under each condition. (a) Base Models. (b) Near Miss Models. (c) SMOTE Models.

The Precision-Recall Curve (PRC) is a valuable metric, especially in imbalanced classification problems like Parkinson’s Disease (PD) detection. It highlights how well the model maintains high precision without sacrificing recall. Fig 11 shows the PRC curves and corresponding average precision scores for all models across the three sampling strategies—Base, SMOTE, and NearMiss. In the Base setting, the KNN model outperformed all others with an average precision of 1.00, followed closely by Random Forest (0.99) and SVM Linear (0.98). XGBoost and SVM RBF both achieved 0.98, showing a strong balance. Other models like Logistic Regression (0.97) and GNB (0.96) also performed reliably, while Decision Tree scored lower at 0.91. Under SMOTE sampling, the average precision scores were consistently higher across the board. Again, KNN and RF achieved perfect precision (1.00), confirming their robustness when trained on balanced data. XGBoost and SVM RBF followed with 0.99, and LR, AdaBoost, and SVM Linear hovered around 0.97, reflecting stability. DT and GNB showed improvement, too, both reaching 0.96. With NearMiss, while some models experienced slight drops in precision, most top performers remained consistent. RF and XGBoost led with 0.99, while KNN, AdaBoost, SVM Linear, and LR each held strong at 0.98. SVM RBF followed with 0.97, and GNB reached 0.93, which is still respectable. Decision Tree lagged at 0.87, highlighting its sensitivity to aggressive undersampling. Models trained with SMOTE showed the most stable and consistently high precision across all classifiers, with KNN standing out as the most reliable model across all three conditions. This is especially important in clinical applications, where reducing false positives is critical to avoid misdiagnosing healthy individuals. The high PRC scores demonstrate the reliability and predictive power of the proposed methods.

**Fig 12:**
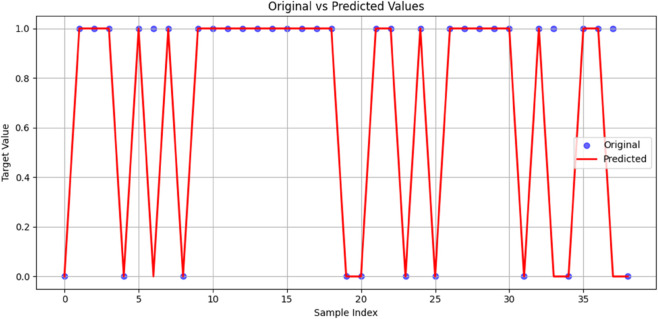
Comparison of original and predicted data demonstrating model effectiveness in capturing patterns.

The binary classifier’s performance was further assessed by contrasting the projected data with the unaltered data. Fig 12 compares the projected data (in orange) with the original data (in blue). The graph demonstrates that the projected data closely matches the real data’s pattern, demonstrating that the classifier successfully identifies the fundamental patterns in the data. The projected data occasionally differs from the original data, indicating that the classifier may need to be more accurate in these circumstances. Comparing the actual and forecasted data shows that the suggested strategy effectively handles the binary classification issue.

### 4.7 XAI visualization

**Fig 13:**
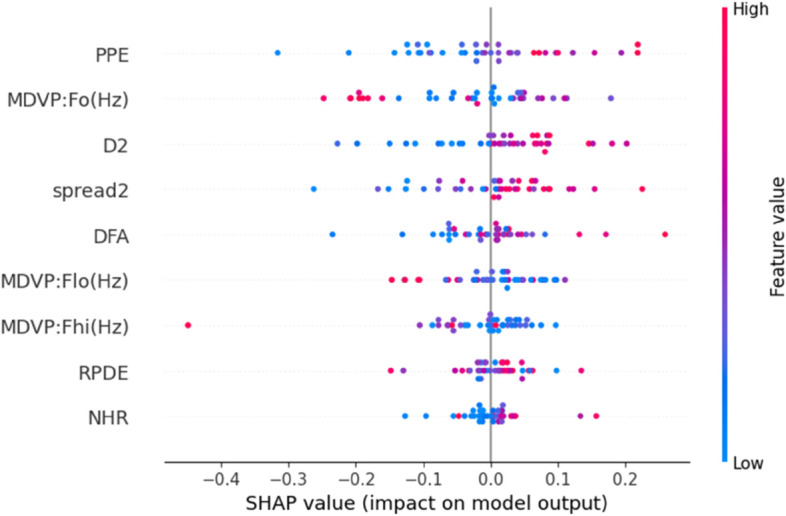
SHAP summary scatter plot showing the effect of individual feature values on the model’s output. Red indicates high feature values, blue indicates low.

**Fig 14:**
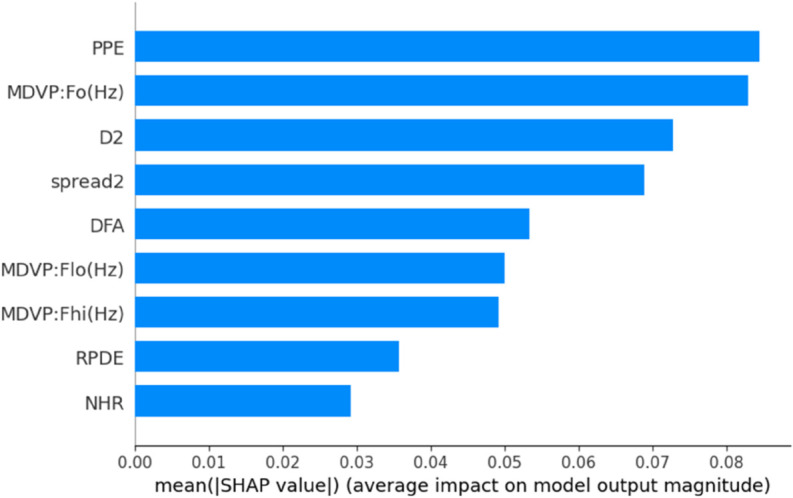
SHAP summary bar plot showing average feature importance. PPE, Fo(Hz), D2, and spread2 were the most influential in the best model.

**Fig 15:**
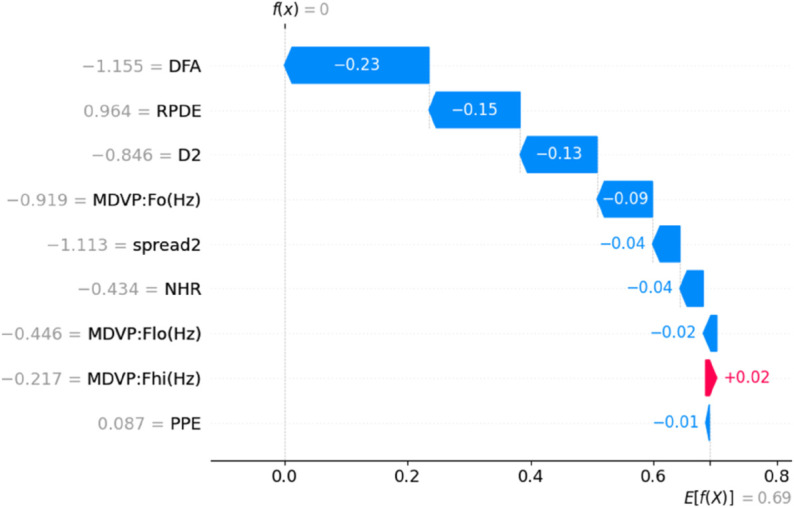
SHAP waterfall plot showing feature-level contributions for a specific prediction. Features such as DFA, RPDE, and Fo(Hz) reduce the prediction score, while PPE slightly increases it.

**Fig 16:**
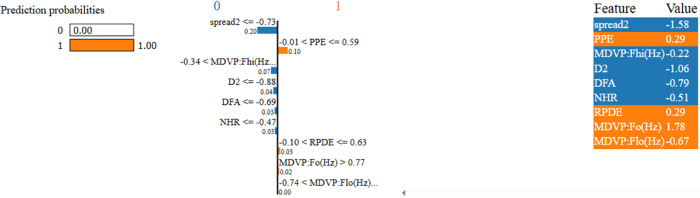
LIME explanation for an individual instance. Features like spread2, PPE, and D2 contribute significantly to the model’s prediction, corroborating the SHAP results.

To enhance the transparency and interpretability of our predictive model, we incorporated Explainable AI (XAI) techniques using both SHAP (SHapley Additive explanations) and LIME (Local Interpretable Model-Agnostic Explanations). After evaluating all nine machine learning models using various data balancing techniques, KNN fine tuned with SMOTE emerged as the top performer with high accuracy, precision, recall, MCC, and Gmean. We applied SHAP and LIME to analyze feature contributions and individual predictions to further understand the model’s decisions. SHAP Analysis: SHAP values offer both global and local interpretability, providing insights into the magnitude and direction of feature impact on model predictions.By showing the initial test results and offering an explanation based on aspects thought to be significant, XAI has made black box models interpretable [37]. It is one of the most widely used techniques today, which uses game theory to explain any ML model’s output [52]. Fig 13 presents the SHAP summary bar plot, revealing that PPE (Pitch Period Entropy) is the most influential feature in the model’s decision-making process, followed closely by MDVP:Fo(Hz), D2, and spread2. This aligns with domain knowledge that these features are strongly associated with Parkinsonian voice disorders. The SHAP scatter plot in Fig 14 further illustrates how individual feature values contribute positively or negatively to the prediction. For instance, higher PPE and Fo(Hz) values tend to increase the likelihood of predicting Parkinson’s Disease. To illustrate the model’s decision on a single instance, we include the SHAP waterfall plot (Fig 15), which visualizes how the combination of feature values pushes the prediction towards or away from the positive PD class. This instance-based reasoning provides critical transparency for clinicians or domain experts. LIME Analysis: Complementing SHAP, we also applied LIME to provide local interpretability. Fig 16 shows the feature influence on a specific prediction using LIME. The explanation is consistent with SHAP findings—spread2, PPE, and D2 are again identified as major contributors to the model’s output. LIME breaks down the decision boundaries using locally linear models, thus offering a human-understandable rationale for the model’s prediction. By integrating both SHAP and LIME, we provide robust, model-agnostic interpretability. These tools collectively enhance the clinical trustworthiness and accountability.

## 5 Conclusion

This study demonstrates the effectiveness of machine learning for improving early diagnosis of Parkinson’s disease. While the highest raw accuracy achieved was 97% using an imbalanced dataset, this performance was likely biased toward the majority class. Our most clinically valuable result was achieved with the KNN classifier using SMOTE, which delivered 92% accuracy, an F1-score of approximately 0.94, and a G-Mean near 0.95—reflecting strong, balanced classification. This trade-off highlights the importance of prioritizing fairness and sensitivity, particularly in healthcare settings. Integrating class balancing techniques (SMOTE and NearMiss) with interpretable machine learning models significantly enhanced both performance and transparency. Feature engineering methods, including chi-square and Featurewiz, effectively identified ten key ten key features (from 23 variables) such as PPE, Fo(Hz), and NHR—contributing to efficient model performance. This approach improves model accuracy and efficiency, which can significantly benefit patient outcomes by enabling earlier, more precise diagnosis. Furthermore, explainable AI tools like SHAP and LIME provided insight into model decisions, reinforcing the clinical relevance of the selected features. Despite using a relatively small secondary dataset, our framework offers a promising, interpretable foundation for PD prediction. Future work will validate this approach with larger, primary clinical datasets and incorporate additional data modalities (e.g., gait, handwriting, or imaging) to improve robustness and generalizability. Ultimately, this research moves a step closer toward developing accurate, fair, and interpretable ML tools suitable for real-world clinical applications.
